# TAS1R1 and TAS1R3 Polymorphisms Relate to Energy and Protein-Rich Food Choices from a Buffet Meal Respectively

**DOI:** 10.3390/nu10121906

**Published:** 2018-12-04

**Authors:** Pengfei Han, Russell Keast, Eugeni Roura

**Affiliations:** 1 Centre for Nutrition and Food Sciences, Queensland Alliance for Agriculture and Food Innovation, The University of Queensland, St. Lucia QLD 4072, Australia; pengfei.han@uq.net.au; 2 Interdisciplinary Centre Smell and Taste, Department of Otorhinolaryngology, Technische Universität Dresden, Fetscherstrasse 74, 01307 Dresden, Germany; 3 School of Exercise and Nutritional Sciences, Centre for Advanced Sensory Science, Deakin University, Burwood VIC, 3126, Australia; russell.keast@deakin.edu.au

**Keywords:** umami taste receptor, single nucleotide polymorphisms, food consumption, buffet meal

## Abstract

Eating behaviour in humans is a complex trait that involves sensory perception. Genetic variation in sensory systems is one of the factors influencing perception of foods. However, the extent that these genetic variations may determine food choices in a real meal scenario warrants further research. This study investigated how genetic variants of the umami taste receptor (TAS1R1/TAS1R3) related to consumption of umami-tasting foods. Thirty normal-weight adult subjects were offered “*ad libitum*” access to a variety of foods covering the full range of main taste-types for 40 min using a buffet meal arrangement. Buccal cell samples were collected and analysed for six single nucleotide polymorphisms (SNPs) reported previously related to the TAS1R1/TAS1R3 genes. Participants identified with the CC alleles of the TAS1R3 rs307355 and rs35744813 consumed significantly more protein from the buffet than T carriers. In addition, participants with GG genotype of the TAS1R1 SNP rs34160967 consumed more fat and calories as compared to the genotype group having the A alleles. In summary, these findings revealed a link between the SNPs variations of umami taster receptor gene and fat and protein intake from a buffet meal.

## 1. Introduction

The impact of single nucleotide polymorphisms (SNP) of chemosensory genes on food intake has been uncovered in recent years [1]. In particular, a few taste receptor SNP have been found associated with food preferences and consumption [2]. For example, sugar intake was found associated with sweet taste receptor (TAS1R2) alleles in humans [3,4,5,6,7]. In addition, the genetic variation of the bitter taste receptor TASR38 has been associated with vegetable, fat and sweet intake [8,9].

Umami (originated from the Japanese word for deliciousness) has been described as the taste of savoury or meaty foods [10]. Umami taste perception requires the stimulation of the heterodimeric G protein-coupled receptor TAS1R1 and TAS1R3, typically by mono-sodium glutamate (MSG) in humans [11,12]. Shigemura [13] reported associations between the genotype variation linked to three common SNPs (rs75881102, rs34160967 and rs307377) of TAS1R1/TAS1R3 and detection thresholds for MSG (umami). It was shown that people having the GG genotype of the TAS1R1 SNP rs34160967 and the CC genotype of the TAS1R3 SNP rs307377 were associated with a decreased MSG threshold [13].

The perception of umami serves as an indicator of protein-rich foods [14]. Despite the known link between TAS1R1/TAS1R3 SNPs variation and umami taste perception, less has been studied on whether this variation is sufficient to influence preferences or intake of protein-rich and umami taste foods [1,15]. In addition, most previous studies regarding taste and food intake used subjective food records or food frequency questionnaires [6,16], with no studies specific for umami taste actually assessing food preference and intake from ad libitum eating sessions. Therefore, the current study investigated the association between allelic variations of selected TAS1R1/TAS1R3 SNPs and food consumption from a buffet meal in a laboratory setting. Since preload sensory exposure prior to the main meal may affect subsequent food choices and intake [17], the current study was designed to determine potential interactions between prior taste exposure and genetic variations, on food or specific nutrient intakes.

## 2. Materials and Methods

### 2.1. Study Participants

Subjects were recruited from the University of Queensland with the following exclusion criteria via questionnaire: history of taste or olfactory dysfunction; smoker; vegetarian; food allergy; drug or medication use; pregnancy, restrained eating score over 14 assessed by the Three Factor Eating Questionnaire (TFEQ) [18]. In addition, participants’ BMI was calculated with self-reported height and body weight. All subjects were asked to give consent for their participation. Thirty adults (14 female) were recruited and completed the study (Table 1). This research protocol was approved by the Queensland University Human Ethical Committee (Ethical approval #2014001036).

### 2.2. Experimental Design

Thirty participants attended three non-consecutive test sessions with a one-week interval in-between, at the food sensory laboratory of the University of Queensland. On each test session, participants arrived between 7:00 a.m. to 9:00 a.m. in a fasted state having consumed only water at least since 11:00 p.m. the night before. First, they were randomly offered one of the three soup preloads which differed in taste: umami; non-umami (with sugar); or no-taste soup (used as an energy-intake control). In each session, the soup preload was different for each participant so that at the end of the three sessions all participants had been exposed to three soup-types. Participants were instructed to eat the soup spoon by spoon and finish the soup within 5 min to ensure a homogeneous oral sensory exposure. One hour later a buffet meal was provided and they were asked to choose any of the foods offered and consume as much or as little as they pleased until comfortably full. Participants were encouraged to try all the food items at the beginning and always ask for more food if the served amount was insufficient. The ad libitum meal session lasted up to 40 min.

### 2.3. Test Foods and Food Intake Calculation

Soup preloads (umami, non-umami or a no-taste) were isocaloric and prepared based on flour and water (60 g plain flour in 1000 g water). Glutamate (MSG, Ajinomoto Co., Inc., Kawasaki, Japan) at 0.5% (*w*/*w*) or sucrose at 1% (*w*/*w*) were added to the soups to elicit the umami and non-umami (sweet) taste, respectively. The sucrose concentration was based on a preliminary pilot study assessing perceivable sweetness and avoiding potential changes of texture that may be perceived by panellists. The soup was prepared fresh and cooled to room temperature before serving. The flour-based soup was selected as it has little to no taste or flavour that may interact with the taste of the preload conditions.

The test buffet meal consisted of four food categories suitable for a morning or midday meal based on predominant tastes (savoury or non-savoury) and the levels of fat content (high vs. low): low-fat sweet, high-fat sweet, low-fat savoury or high-fat savoury foods. High fat foods were defined as a minimum 25% (*w*/*w*) fat content. Two food items were selected to be offered as choices for each of the four categories. All foods served were provided in excess of the estimated energy requirements (three times the suggested serving size). Menus, nutritional information and serving size of each food item are shown in Table 2. 

Each food item was weighted before and after the ad libitum eating session. Total carbohydrate, protein, fat, sodium and energy intakes were calculated from the nutritional information supplied in the label of the commercial products. In addition, the sensory-based food choices and consumption were measured by combining the amount of food consumed from food items classified as “sweet” or “savoury”. The intake of the following food categories and nutrients were included for analysis: total energy (kJ), carbohydrate, protein, fat or sodium (in grams and as % of total energy); sweet foods (including the sum of intake of low or high-fat sweet food categories); savoury foods (including the sum of the intake of low or high-fat savoury food categories).

### 2.4. Gene Sequencing

Oral cell samples were collected using the Gentra Puregene Buccal Cell Kit (Qiagen, Germantown, MD, USA) and DNA was extracted following the protocol recommended by the supplier. Target SNPs were selected with two criteria: (1) involving the taste receptor genes (TAS1R1/TAS1R3) previously reported to be associated with umami taste perception [13,19,20,21]; and (2) the allele frequency of the SNP in the population was >10% according to previous literature and the genetic database [4]. Six target SNPs were selected: 3 TAS1R1 SNPs (rs41278020, rs34160967 and rs35118458) and 3 TAS1R3 SNPs (rs307355, rs307377 and rs35744813). The method of SNP genotyping was validated by the Australian Genome Research Facility (AGRF). Target DNA was amplified using custom-designed PCR primers in a final reaction volume of 5 µL, PCR conditions were 95 °C for 2 min, followed by 45 cycles of 95 °C for 30 s, 56 °C for 30 s, 72 °C for 1 min and 72 °C for 5 min and finally a holding temperature of 4 °C. Individual SNP genotyping was performed using the MassARRAY system and TyperAnalyzer from Agena Bioscience (Hamburg, Germany).

### 2.5. Statistical Analysis

Food consumption across the whole cohort (*n* = 30) was compared between preload taste type using one-way repeated measures analysis of variance (ANOVA). The two-way repeated-measures ANOVA was used to examine the differences in food consumptions from the buffet meal with preload conditions (umami, sweet, or non-taste control soup) as within-subject factor, with SNP variations as the between-subject factor, including body weight, age and gender as covariates. Post-hoc pairwise comparisons, corrected for multiple comparisons using the Bonferroni method, were performed when ANOVA analyses were significant. Pearson’s correlations were used to determine associations between variables. Data analysis was performed using SPSS 20.0 (SPSS Inc., Somers, NY, USA). Data was presented as mean ± Standard Error of the Mean and differences were considered significant at *p* < 0.05 and as a trend at *p* < 0.1.

## 3. Results

### 3.1. TAS1R1/TAS1R3 SNP Sequencing Result

Table 3 summarizes the sequencing results for the selected SNPs of TAS1R1 and TAS1R3. We studied three SNP locus related to the TAS1R1: the rs41278020, rs34160967 and the rs35118458. The CC allele of the rs41278020 (27 out of 28 participants) and GG allele of rs35118458 (all participants) were dominant, while for rs34160967, 14 subjects were identified with the GG allele, 10 subjects with GA allele and 3 subjects with AA allele. Regarding the TAS1R3 the allelic frequency was identical for rs307355, rs307377 and rs35744813, consisting of one predominant allele (CC) with 75% frequency and two minor alleles (TC and TT) accounting for the rest (Table 3). In addition, our results contained several sequencing errors which resulted in discarding two participants for the rs41278020, three participants for rs34160967, three for rs35744813 and one for rs307355 and rs307377.

### 3.2. SNP Variations and Food Consumption

Table 4 shows the averaged food consumption in all the study participants. The repeated ANOVA analyses found no significant effect of the preload condition on energy or food consumptions (Table 4, all *p* > 0.1).

The genotype-phenotype relations regarding the SNPs of interest were assessed when the two homozygotic genotypes were identified in the cohort of subjects. That was the case for three SNPs: the TAS1R1 rs34160967 (GG or AA) and the TAS1R3 rs307355 and rs35744813 (both CC and TT). 

For the three SNPs of interest, the repeated-measures ANOVA showed no main effect due to the preload condition, or interaction of preload with genetic variations (all *p* > 0.1). Therefore, dietary intake was analysed comparing different SNP variations (Table 5). Due to the small sample size and limited number of minor allele carriers, participants with either homozygous (AA for rs34160967; TT for rs307355 and rs35744813) or heterozygous (CA for rs34160967; CT for rs307355 and rs35744813) were merged into one group. 

For the TAS1R3 gene, individuals presenting the CC haplotype at locus rs307355 and rs35744813 consumed significantly more protein from the buffet meal than the T carriers (including the heterozygotes CT or TC or homozygotes TT). In addition, TT carriers tended (*p* = 0.05) to consume more savoury foods from the buffet compared to C carriers at locus rs35744813 (Table 5). For the TAS1R1, the GG compared to A carriers at rs34160967 consumed significantly more energy and higher amount of fat from the buffet meal (*p* = 0.03, Table 5).

Total energy consumption was correlated to the amount of savoury food eaten (*r* = 0.61, *p* < 0.001 for savoury preload; *r* = 0.70, *p* < 0.001 for non-savoury preload; *r* = 0.69, *p* = 0.001 for non-taste control preload), however, weak correlation was observed between energy intake and amount of sweet food eaten (*r* = 0.33, *p* = 0.08 for savoury preload; *r* = 0.35, *p* = 0.06 for non-savoury preload; *r* = 0.47, *p* < 0.01 for non-taste control preload).

The other three SNPs (rs41278020, rs35118458 and rs307377) were not analysed for food consumptions given the small group of participants in the non-predominant haplotype.

## 4. Discussion

The study used overnight fasting and breakfast consisting of isocaloric soup preloads to account for energy intake and taste exposure prior to the test meal. Preload taste exposure (umami vs. non-umami) had no influence on subsequent food choices. Importantly, the results showed that selected SNP variations of the umami taste receptor gene (TAS1R1/TAS1R3) were associated with energy or protein-rich/savoury food consumption in a laboratory-setting buffet meal. 

TAS1R1 gene GG carriers at rs34160967 consumed more fat and energy compared to the A carriers. It was known that the variation is associated with umami taste perception [21]. One explanation is the interaction between umami and other taste modalities. For example, the rs34160967 AA/AG genotype group reported lower intensities than GG genotype group for oral perception of salty (NaCl), sweet (sucrose), sourness (citric acid) and bitterness (Quinine hydrochloride) [22]. A possible interaction between umami and fat taste is also possible. In addition, fat sensation has been closely related to umami taste and saltiness in commonly consumed foods [14,23]. Our results suggest that dietary fats have the potential to interact with the salty or umami taste and increase the food palatability. This was also supported by the significant correlation between total energy and savoury food consumptions. In addition, the interaction between fat and the TAS1R1 seem to be influenced by the mutation in the locus rs34160967. Thus, rs34160967 polymorphisms may have a direct impact on the perception of dietary fats linked to the umami taste receptor subunit TAS1R1. A previous study on the same SNP suggested that the AG genotype group consumed more umami-food as compared to GG carriers [16]. A direct comparison between the current study with Choi [16] is difficult. First, Choi et al. [16] only included female subjects and it is possible that, compared to our study, the gender difference could have delivered a different result related to a gender effect [24]; and second, different measurements of food intake were used, which may also explain some of the discrepancies. 

TAS1R3 gene CC carriers compared to TC or TT carriers at rs307355 and rs35744813 consumed a larger amount of protein and savoury-tasting food from the buffet (the latter being a trend only). Umami taste indicates high protein content in common foods [14,25] and correlations between umami taste sensitivity and umami preference or dietary protein intake has been suggested (Pepino et al., 2010; Luscombe-Marsh et al., 2008). The inter-individual differences for umami thresholds may be as high as five hundred-fold between high and low sensitivity subjects [26]. In addition, our results were in line with others showing umami taste differences related to genetic variations of the TAS1R3 [13,19,20]. Thus, our data supports the direct role of the umami taste receptor stimulation (i.e., TAS1R3) in modulating dietary protein appetite.

It is important to note that the TAS1R3 protein is shared by the sweet and umami heterodimer complexes. Variation of allelic polymorphism of the TAS1R3 have also been related to sweet taste perception with the preference for higher concentration of sucrose in water among T compared to the CC haplotypes of rs35744813 [27,28]. Others have reported the TAS1R3 SNP rs307355 to be related to alcohol consumption [29]. However, to date, there has been no report associating these two selected SNPs and specific food appetite or consumption. 

The current study had some limitations worth noting. First, the SNP on the genes of interest studied were selected to those known to be involved in food intake or taste perception, while several others were discarded. However, it is possible that other allelic variations may have an impact on the function and/or expression of the genes of interest. This, in turn, could affect food behaviour to a similar or greater extent than those SNP reported here. Second, food intake was observed within one eating session and with limited number of food items; third, environmental and cultural factors (such as the participants’ childhood eating/dietary habits) can confound how gene polymorphisms impact on dietary intake [21,30], which suggests unmeasured environmental factors may partially explain or swamp the genetic effect; Finally, the authors acknowledge that the sample size was small for a genetic association study and the findings need to be interpreted with caution until corroborated with further research with a larger sample size.

## 5. Conclusions

In conclusion, the study reports that TAS1R1 and TAS1R3-linked polymorphic genotypes at rs34160967 and rs307355 and rs35744813, respectively, are associated with fat and savoury-tasting and protein-rich food choices following a laboratory buffet in humans. To our knowledge, this is the first study that showed genetic variants of the umami taste receptor involved in differential food choices.

## Figures and Tables

**Table 1:** Participants’ characteristics.

Parameter	*n* = 30		
	Range	Mean	SEM
Age (years)	20~37	27.4	0.69
BMI (kg/m^2^)	19.1~25.7	22.5	0.32
TFEQ Restraint	2~14	7.3	0.68
TFEQ Disinhibition	2~13	6.3	0.54
TFEQ Hunger	0~14	4.8	0.60

SEM, Standard Error of the Mean; BMI, Body Mass Index; TFEQ, Three Factor Eating Questionnaire.

**Table 2:** Nutritional information for food items included in the buffet meal ^1^.

Classification	Food Item ^2^	Predominant Nutrient(s)	Calorie	Carb	Protein	Fat/Sat. Fat	Sodium	Serve ^3^	Serve Calorie
			(kJ)	(g)	(g)	(g)	(mg)	(g)	(kJ)
Low-fat sweet	Apple	Carb	218	13.81	0.26	0.17/NA	1	100	218
	Banana	Carb	372	22.84	1.1	0.33/NA	1	200	744
	Melon	Carb	151	9.1	0.54	0.14/NA	16	250	375
	Fruit Loaf	Carb/Sodium	1510	66.8	10.5	4.5/0.8	370	150	2265
High-fat sweet	Muffin	Carb/Fat/Sodium	1920	57	5	23.1/4	300	135	2592
	Shortbread	Carb/Fat/Sodium	2210	58.4	5.6	30.3/18.9	277	66	1459
Low-fat savoury	Tomato	Carb	75	3.9	0.9	0.2/NA	5	150	113
	Green pepper	Carb	84	4.64	0.86	0.17/NA	7	100	84
	Carrot	Carb	172	10	1	0.24/NA	69	100	150
	Ham	Protein/Sodium	364	1.3	14.9	2.4/0.8	790	150	546
High-fat savoury	Nuts	Protein/Fat/Sodium	2885	13.4	22.7	54.6/7.9	275	100	2885
	Cheese	Protein/Fat/Sodium	1720	1	24.8	34.7/23.8	650	66	1135

^1^ Energy and nutrient content calculations used manufactures’ package information; ^2^ Apple: Coles Pink Lady Apple; Banana: Coles banana; Melon: Coles Rock Melon; Fruit Loaf: Tip Top café raisin toast; Muffin: Coles Chocolate chip Muffin bars; Shortbread: Walkers Cello Shortbread Finger; Tomato/Green pepper/Carrot: Fresh from Coles; Sliced Ham: Coles English Ham; Nuts: Coles Salted Mixed Nuts; Cheese: Beqa tasty cheese slices. ^3^ Initial serving size; Abbreviations: Sat., Saturated; Carb, Carbohydrate.

**Table 3:** Allelic distribution for selected SNP expressed as proportion of participants (%).

Gene	SNP	Polymorphisms	Overall (*n*)	Proportion of Participants (%)
TAS1R1	rs41278020	CC	27	96.4
		CT	1	3.6
		TT	0	-
	rs34160967	GG	14	51.9
		GA	10	37.0
		AA	3	11.1
	rs35118458	GG	29	100
		GA	0	-
		AA	0	-
TAS1R3	rs307355	CC	22	75.9
		TC	5	17.2
		TT	2	6.9
	rs307377	CC	25	86.2
		TC	4	13.8
		TT	0	-
	rs35744813	CC	20	74.1
		TC	5	18.5
		TT	2	7.4

Overall percentage indicate the percentage of frequency for each polymorphism in the current study population.

**Table 4:** Effect of pre-load treatment group (umami, non-umami and control) on the consumption of foods from a buffet meal categorised by macro-nutrient (energy, carbohydrate, protein or fat), taste category (sweet or savoury) or energy contribution (%) (*n* = 30 participants).

Food Category	Umami Preload	Non-Umami Preload	Control Preload
Total energy (kJ)	4055.6 (342.7)	3735.6 (329.3)	3937.1 (313.6)
Sweet food (g)	332.1 (21.7)	342.6 (22.5)	332.2(23.5)
Savoury food (g)	205.3 (25.3)	197.9 (25.7)	209.8 (25.3)
Total Carb (g)	106.8 (8.3)	99.4 (8.3)	104.0 (7.9)
Total Prot (g)	32.2 (3.3)	30.6 (3.3)	31.9 (3.3)
Total Fat (g)	45.3 (5.2)	40.9 (4.9)	43.6 (4.2)
% Energy Carb	45.4 (2.2)	46.4(2.4)	45.0 (1.7)
% Energy Prot	13.2 (0.7)	13.2 (0.6)	13.2 (0.8)
% Energy Fat	41.1 (1.8)	40.1 (2.0)	41.1 (1.3)

Data are shown as Mean (SEM); % Energy Carb, carbohydrate as percentage of energy; % En Prot, protein as percentage of energy; % En Fat, fat as percentage of energy.

**Table 5:** Effect of umami taste receptor (TAS1R1/TAS1R3) allelic genotypes on the consumption of foods from a buffet meal, categorised by macro-nutrient (energy, carbohydrate, protein or fat), taste category (sweet or savoury) or energy contribution (%) (*n* = 30 participants).

Food Category	TAS1R1			TAS1R3					
	rs34160967			rs34160967			rs34160967		
	GG (*n* = 14)	GA/AA (*n* = 13)	*p* value	CC (*n* = 22)	CT/TT (*n* = 7)	*p* value	CC (*n* = 20)	CT/TT (*n* = 7)	*p* value
Energy (kJ)	4045.2 (315.9)	3751.1 (316.1)	0.03	4184.4 (332.7)	3055.2 (600.8)	0.12	4272.2 (359.2)	3047.6 (618.2)	0.11
Sweet food (g)	347.3 (25.8)	308.4 (16.8)	0.32	338.7 (20.0)	309.6 (36.0)	0.49	345.9(21.2)	308.6(36.4)	0.39
Savoury food (g)	218.2 (31.0)	170.6 (32.2)	0.31	226.4 (23.7)	131.7 (42.7)	0.07	228.1 (23.9)	128.3(41.1)	0.05
Total Carb (g)	112.8 (9.1)	89.7 (9.4)	0.10	105.3 (7.2)	93.2 (13.0)	0.43	107.0 (7.8)	93.1 (13.5)	0.39
Total Prot (g)	35.4 (4.0)	26.8 (4.1)	0.16	34.8 (3.0)	21.4 (5.3)	0.04	35.6 (3.1)	21.2 (5.4)	0.03
Total Fat (g)	54.9 (6.4)	33.3 (6.6)	0.03	21.4 (5.3)	48.2 (5.3)	0.11	43.4 (5.7)	29.9 (9.8)	0.11
% Energy Carb	43.8 (2.8)	45.7 (2.9)	0.64	43.9(2.2)	49.1 (3.9)	0.26	43.4 (5.7)	49.2 (4.1)	0.27
% Energy Prot	12.7 (1.0)	13.4 (1.0)	0.66	13.6 (0.7)	12.0 (1.4)	0.31	13.7 (0.8)	11.9 (1.4)	0.28
% Energy Fat	42.7 (2.2)	40.9 (2.3)	0.57	42.2 (1.7)	38.5 (3.1)	0.32	42.3 (1.9)	38.6 (3.2)	0.34

Data are shown as Mean (SEM); numbers in the bracket after different polymorphism indicate the number of participants; % Energy Carb, carbohydrate as percentage of energy; % Energy Prot, protein as percentage of energy; % Energy Fat, fat as percentage of energy; Carb: carbohydrate; Prot: protein; t; *p* value indicates the main effect of genetic variation (different SNP polymorphisms) using repeated ANOVA.
